# Synergistic Bioconversion of Date Palm Leaves Into Ruminant Feed by a Leopard Moth ( *Zeuzera pyrina* L.) Gut Bacterial Consortium and Nutrient Stimulators

**DOI:** 10.1002/fsn3.71461

**Published:** 2026-02-02

**Authors:** Afrooz Sharifi, Ayoub Azizi, Ali Kiani

**Affiliations:** ^1^ Animal Science Research Department Khuzestan Agricultural and Natural Resources Research and Education Center, Agricultural Research, Education and Extension Organization (AREEO) Ahvaz Iran; ^2^ Department of Animal Science, Faculty of Agriculture Lorestan University Khorramabad Iran

**Keywords:** bacteria consortium, biological delignification, in vitro fermentability, leopard moth, nutritive value

## Abstract

Date palm leaves (DPL), a widely available lignocellulosic by‐product, are used as ruminant feed but are limited by high lignin and low protein content. This study evaluated the enhancement of DPL's nutritional value using a lignocellulose‐degrading bacterial consortium (*Staphylococcus* sp., *Brevibacterium* sp., and *Enterobacter* sp.) isolated from the leopard moth ( *Zeuzera pyrina* L.) gut, supplemented with microbial growth stimulators. Six treatments were applied: untreated DPL (control), DPL with M9 medium (T1), DPL with bacterial inoculum (T2), T2 + 0.5% glucose (T3), T2 + 0.5% urea (T4), and T2 + 0.5% glucose + 0.5% urea (T5). Parameters assessed included chemical composition, lignin peroxidase (LiP) activity, in vitro gas production (IVGP), fermentation characteristics, nutrient digestibility, and ruminal enzyme activities. LiP activity was highest in T5 (0.328 U/mL/min), representing a significant increase over controls. Inoculated treatments significantly reduced acid detergent lignin (ADL) and increased crude protein (CP), with T5 showing the greatest improvement: ADL decreased from 97.8 to 83.8 g/kg DM, and CP increased from 52.2 to 70.3 g/kg DM. T5 also exhibited the highest dry matter (DM) loss (61.1 g/kg DM), IVGP (61.2 mL), metabolizable energy (5.61 MJ/kg DM), short‐chain fatty acid concentration (2.43 mmol/g DM), microbial protein synthesis (405 mg/g DM), and ammonia‐N (10.2 mg/dL). Activities of carboxymethyl cellulase, microcrystalline cellulase, and filter paper‐degrading enzymes were significantly elevated in all inoculated treatments, with T5 consistently yielding the highest values. These results demonstrate that co‐application of the leopard moth gut‐derived bacterial consortium with glucose and urea effectively delignifies DPL, substantially enhances its fermentability and nutritive value, and offers a sustainable strategy for valorizing agricultural residues in ruminant nutrition.

## Graphical abstract

Treating date palm leaves with a bacterial consortium, especially when supplemented with glucose and urea, significantly degrades lignin. This process enhances nutritional value by increasing crude protein, digestibility, and metabolizable energy. The method effectively transforms this agricultural by‐product into a viable ruminant feed.

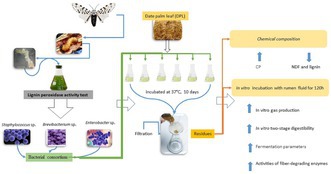

## 1 Introduction

Given the rising global demand for livestock products, and the increasing cost of conventional animal feed, it is essential to utilize alternative cost‐effective and nutritionally adequate feed resources (Jami et al. 2015; Malenica et al. 2023; Kazemi 2025). Agricultural by‐products already constitute a significant portion of animal diets, and their nutritional value can be further enhanced through various processing techniques (Malenica et al. 2023; Alimi et al. 2024). Among these resources, lignocellulosic by‐products such as date palm leaves (DPL) are widely available in many regions. These materials are predominantly composed of structural carbohydrates (i.e., cellulose, hemicellulose, and pectin) and are characterized by a high content of a phenolic compound of lignin (Kusmiati et al. 2024; Zhong et al. 2021). However, the high lignin concentration and low crude protein (CP) content inherently limit the digestibility and the effective utilization of lignocellulosic material in ruminant diets (Khatoon et al. 2022; van Kuijk et al. 2015). This recalcitrance is primarily due to lignin forming a complex matrix with structural carbohydrates, which impedes microbial degradation under anaerobic conditions in the rumen (Kusmiati et al. 2024; Zheng et al. 2025).

To improve the feed value of lignocellulosic by‐products, various pretreatment methods including mechanical, chemical, thermal, and biological processes have been developed (Alavijeh et al. 2020; Amin et al. 2017). Nevertheless, the practical application of many of these methods is often constrained by high operational costs, substantial energy demands, technical complexity, or adverse environmental impacts (Zheng et al. 2025). As promising an alternative, biological pretreatment using lignocellulolytic microorganisms has emerged as a sustainable strategy for improving the digestibility of fibrous agricultural residues (Azizi‐Shotorkhoft et al. 2016; Kusmiati et al. 2024). Among these microbial agents, bacteria that secrete cellulolytic and ligninolytic enzymes such as cellulases, xylanases, and ligninases are of particular interest for deconstructing plant cell wall into more accessible and fermentable forms.

In nature, the efficient decomposition of plant biomass is exemplified by insects such as termites, beetles, and cockroaches. This capability is largely attributed to the presence of specialized digestive enzymes and symbiotic microorganisms within their gastrointestinal tracts (Azizi‐Shotorkhoft et al. 2016; Tartar et al. 2009). The leopard moth ( *Zeuzera pyrina* L., Lepidoptera: Cossidae), a globally distributed wood‐boring insect, is a notable example. Adult leopard moths generally deposit their eggs in young, developing tree shoots. Once the larvae emerge, they begin boring into the branches and feeding on the internal wood. Its larvae feed exclusively on lignified plant tissues for 2–3 years, progressing from smaller branches to the main trunk (Kutinkova et al. 2006; Langström et al. 2004). The remarkable ability to digest woody lignified plant tissues suggests the presence of lignocellulose‐degrading microorganisms within its gut. To date, the microbial communities inhabiting the digestive tract of the leopard moth, and their potential application in bio‐converting fibrous crop residues into ruminant feed, remain unexplored.

The efficacy of microbial inoculants depends not only on their enzymatic potential but also on their mode of application. While single bacterial strain inoculants can be effective for targeted substrate breakdown, they often lack the synergistic interactions required for the complete degradation of complex lignocellulosic structures. In contrast, microbial consortia, comprising functionally diverse strains, can emulate robust and synergistic dynamics of natural ecosystems like the human or insect gut. Such consortia typically exhibit greater metabolic versatility, stability, and efficiency in degrading a wider range of lignocellulosic compounds (Coelho et al. 2025; Zhou et al. 2018). Furthermore, supplementing culture medium with readily available carbon and nitrogen sources, such as glucose and urea, has been shown to enhance microbial proliferation and enzymatic activity (Borji 2003). The synergistic use of these additives may therefore amplify microbial production and the enzymatic breakdown of fibrous components.

The present study focuses on a bacterial consortium of *Staphylococcus* sp., *Brevibacterium* sp., and *Enterobacter* sp., which were isolated from the gut of leopard moth via 16S rRNA gene sequencing (Azizi et al. 2021). To our knowledge, no prior research has investigated the application of a bacterial consortium derived from the leopard moth gut for the bioconversion of agricultural by‐products. Therefore, this study aimed to investigate the synergistic effects of this lignocellulose‐degrading bacterial consortium, with and without microbial growth stimulators (glucose and urea) on enhancing the nutritional value of DPL for ruminant feeding. The effects were assessed based on changes in chemical composition, in vitro gas production (IVGP), fermentation characteristics, and microbial enzymatic activity as key indicators of improved feed value.

## 2 Materials and Methods

### 2.1 Sample Collection and Site Description

DPLs were collected during the summer of 2024 from various agricultural locations near Ahvaz city (approximately 31°20′ N, 48°40′ E) in Khuzestan Province, southwestern Iran. This region lies within the Khuzestan Plain, an alluvial lowland of the Tigris‐Euphrates and Karun river systems, bordering the Mesopotamian basin to the west. The area features calcareous alluvial soils (Aridisis order) with a silty clay loam to clay loam texture. These soils are typically alkaline (pH > 7.5) and moderately to highly saline, conditions to which the date palm is well‐adapted.

The climate is arid, classified as BWh (hot desert climate) under the Köppen–Geiger system. Summers are prolonged and extremely hot, with average maximum temperatures often surpassing 45°C in July and August. Winters are mild (8°C–18°C) and the region receives scant, erratic precipitation, with an average annual rainfall below 200 mm occurring mainly from November to April. Relative humidity is frequently high, exceeding 70% in summer, particularly along the coastal plain.

### 2.2 Leaf Sampling and Processing

Samples of DPL were obtained from four distinct agricultural sites. At each location, mature, undamaged leaves were collected from five healthy date palm trees (approximately 10–14 years of age), resulting in a total of 20 samples. Using sterilized equipment, the leaves were cut, placed in clean plastic bags, and transported immediately to the laboratory. In the lab, the leaves were first thoroughly dried and ground using a Wiley mill (Ogaw Seiki Co. Ltd., Tokyo, Japan) equipped with a 1 mm sieve. To remove soluble compounds that could act as confounding variables in the assessment of growth stimulators (Borji 2003; Azizi‐Shotorkhoft et al. 2016), the ground material was subjected to a hot water extraction process with three successive water changes over 1 h. The resulting insoluble fibrous residue was dried at 60°C for 48 h and subsequently incorporated into the growth medium as the experimental growth substrate.

### 2.3 Bacteria Isolation, Screening and Identification

**FIGURE 1:**
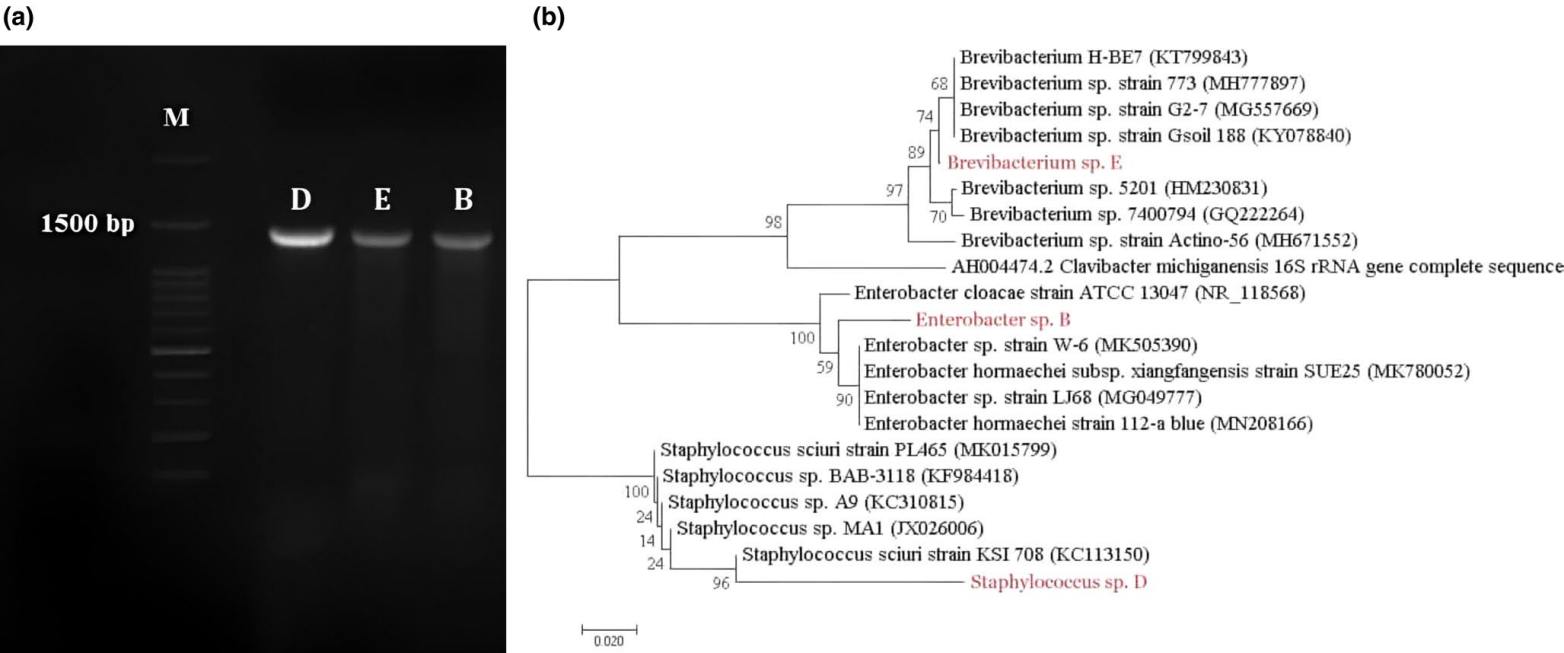
PCR products of isolates D, E, and B on agarose gel (a), and phylogenetic tree of *Staphylococcus* sp. (D), *Brevibacterium* sp. (E), and *Enterobacter* sp. (B) isolated from *Zeuzera pyrina* L. gut based on neighbor‐joining method (b).

The bacterial strains used in this study were previously isolated from the hindguts of the leopard moth, *Zeuzera pyrina* L. (Azizi et al. 2021). Briefly, third instar larvae (under 1 year of age) were collected from infected walnut branches located at Saman County of the Chaharmahal and Bakhtiari Province in the West of Iran, and their body surfaces were sterilized by immersion in 95% ethanol followed by washes in sterile Terrific Buffer (TB) medium (Kato et al. 1998). Hindguts were dissected, and their contents were homogenized in TB medium supplemented with 300 mg/L of dealkalized lignin to create a gut inoculum. For isolation, 1 mL of the gut inoculum was transferred to Erlenmeyer flasks containing sterile basal media (SBM) supplemented with 0.5 g/L of various lignocellulosic substrates including wheat straw (WS), HCl‐lignin of WS, sawdust, HCl‐lignin of sawdust, sugarcane tops (ST), HCl‐lignin of ST, and kraft lignin. The SBM contained the following ingredients per liter: 7.0 g K_2_HPO_4_, 3.0 g KH_2_PO_4_, 1.0 g (NH_4_)_2_SO_4_, 0.1 g MgSO_4_·7H_2_O (Cartwright and Holdom 1973). After incubation at 30°C for 48 h with shaking, cultures were spread onto corresponding agar plates. Distinct bacterial colonies were purified through successive sub‐culturing, yielding eight unique isolates. These isolates underwent a two‐stage screening process. First, they were assessed for their ability to proliferate on seven different media containing lignin and lignocellulose. The five isolates that passed this initial screen were then screened for their functional capacity to degrade lignin by measuring lignin peroxidase (LiP) activity (Archibald 1992). This assay involved culturing the isolates in M9 medium with kraft lignin and quantifying the enzyme's ability to oxidize Azure B dye. Three isolates exhibiting the highest LiP activity were selected for molecular identification. Genomic DNA was extracted from each isolate, and the 16S rRNA gene was amplified via polymerase chain reaction (PCR) using universal primers (Ausubel et al. 1992). The PCR reaction mixture (total volume 25 μL) comprised 1.0 μL of template DNA, 1.5 μL of Taq DNA polymerase, 2.5 μL of 10X buffer, 200 μM of each dNTP, 1 μL of forward and reverse primers (each at 10 ρmol concentration), 2 mM MgCl_2_, and deionized water. The amplification was carried out under the following cycling conditions: an initial denaturation step at 94°C for 5 min; 30 cycles of denaturation at 94°C for 1 min, annealing at 58°C for 40 s, and extension at 72°C for 150 s; followed by a final extension at 72°C for 20 min. The resulting PCR products were visualized on a 1% agarose gel stained with DNA safe stain (Figure 1a). Subsequently, the PCR amplicons were sequenced and compared to sequences in the GenBank database via the BLAST algorithm at the National Center for Biotechnology Information. Phylogenetic analysis identified the three strains as members of the genera *Staphylococcus*, *Brevibacterium*, and *Enterobacter* (Figure 1b). These identified strains were then cultured to prepare the bacterial inoculum mixture for the DPL treatment experiments.

### 2.4 Experimental Treatments and Inoculum Preparation

A liquid culture M9 medium, formulated according to Kato et al. (1998), was used as the base for all cultures. The medium contained the following per liter: 6.2 g disodium hydrogen phosphate (Na_2_HPO_4_), 3.0 g potassium dihydrogen phosphate (KH_2_PO_4_), 0.5 g sodium chloride (NaCl), and 1.0 g ammonium chloride (NH_4_Cl). Nutrient supplement (glucose and urea) was added as specified in the treatments. The bacterial inoculum consisted of a combination of three lignocellulose‐degrading bacterial strains (*Staphylococcus* sp., *Brevibacterium* sp., and *Enterobacter* sp.), originally isolated from the gut microbiota of the leopard moth. Each strain was cultured individually in nutrient broth for 48 h, and the cell density was enumerated to a final concentration of 10^7^ CFU/mL for each, as determined by the method of Botaro et al. (2013). The nutrient broth contained 5.0 g beef extract, 5.0 g peptic digest of animal tissue, 1.50 g sodium chloride, and 1.50 g yeast extract in 1 L of distilled water (Saha and Paul 2014). The mixed inoculum was prepared by combining equal volumes (1 mL each) of these three bacterial suspensions. Six experimental treatments were established: an untreated DPL (control), DPL with M9 liquid medium (T1), DPL treated with 3 mL of the mixed bacterial inoculum, T2 + 0.5% glucose (T3), T2 + 0.5% urea (T4), and T2 + 0.5% glucose + 0.5% urea (T5).

### 2.5 LiP Activity Assay

LiP activity was determined for all six treatments using a modified version of the procedure described by Archibald (1992). For each treatment, five replicate flasks (100 mL Erlenmeyer type) were prepared (30 flasks in total). Each flask contained 10 mL of M9 medium and 100 mg of water‐extracted DPL as the sole carbon source (1% w/v final concentration). Each flask was inoculated with 3 mL of the prepared mixed bacterial suspension. The cultures were incubated at 37°C with continuous shaking at 110 rpm for 8 days. Following incubation, crude enzyme extracts were prepared according to the method of Arora and Gill (2001). The culture contents were first filtered through Whatman No. 1 filter paper to remove particulate matter. The filtrate was then centrifuged at 10,000 × *g* for 20 min, and the resulting clear supernatant (CS) was collected as the crude enzyme extract. LiP activity was assessed by measuring the oxidative de‐colorization of Azure B. The reaction mixture contained 1.0 mL of 125 mM sodium tartrate buffer (pH 3.0), 500 μL of 0.16 mM Azure B dye, 500 μL of the crude enzyme extract, and 500 μL of 2 mM hydrogen peroxide. The reaction was initiated by the addition of hydrogen peroxide, and the decrease in absorbance was monitored at 610 nm. One unit of LiP activity was defined as a reduction in optical density by 0.1 per min per mL of culture filtrate. The resulting enzyme activity levels for all treatments are presented in Table 1.

**TABLE 1:** Lignin peroxidase activity (U/mL/min) of leopard moth gut bacterial mixture (i.e., *Staphylococcus* sp., *Brevibacterium* sp., and *Enterobacter* sp.) cultured on date palm leaf (DPL) with different feed additives (*n* = 5).

Experimental treatments	Activity
Control	0.00^d^
T1	0.00^d^
T2	0.202^c^
T3	0.269^b^
T4	0.266^b^
T5	0.328^a^
SEM	0.009
*p*	< 0.01

*Note:* In column, values followed by different letters are significantly different (*p* < 0.05). The experimental treatments included control (untreated DPL), T1 (DPL + M9 liquid medium), T2 (DPL + bacterial inoculum comprising a mixture of *Staphylococcus* sp., *Brevibacterium* sp., and *Enterobacter* sp.), T3 (T2 + 0.5% glucose), T4 (T2 + 0.5% urea), and T5 (T2 + 0.5% glucose +0.5% urea).

Abbreviation: SEM, standard error of the mean.

### 2.6 Biological Processing of DPL

A total of 30 1 L Erlenmeyer flasks were used to implement six different treatments, with each treatment replicated five times. Each flask contained 500 mL of M9 medium, supplemented with 2.5% (w/v) of water‐extracted DPL, equaling 12.5 g per flask. After sterilization by autoclaving, the flasks were inoculated with 3 mL bacterial suspension. This inoculum consisted of equal volumes (1 mL each at 10^7^ CFU/mL) of *Staphylococcus* sp., *Brevibacterium* sp., and *Enterobacter* sp., which had been pre‐cultured in nutrient broth for 48 h. The flasks were vortexed for 2 min to ensure the uniform distribution of the inoculum across the DPL substrate. Subsequently, all flasks were incubated under aerobic conditions at 37°C in a shaking incubator (110 rpm) for 21 days. After the incubation period, the culture contents were filtered to separate the solid residue. The recovered solids were oven dried and weighed. For further analytical procedures, the dried residues from four of the five replicates per treatment were combined into a single sample.

### 2.7 Rumen Content Sampling and In Vitro Fermentation Procedures

Rumen fluid was collected from two adult, rumen‐cannulated Lori rams (average body weight: 45.0 ± 1.14 kg) housed at the Agricultural Research Station of the Faculty of Agriculture, Lorestan University. The animals were kept under controlled conditions and all experimental procedures conducted in strict compliance with animal welfare standards, reviewed and approved by the institutional guidelines based on the Guide for the Care and Use of Agricultural Animals in Research and Teaching (FASS 2010). The rams were fed a balanced total mixed ration (TMR), formulated to meet their maintenance requirements according to NRC (2007) recommendations. The dry matter (DM) composition of the TMR (g/kg DM) was as follows: WS (400), alfalfa hay (100), corn silage (100), ground corn grain (270), wheat bran (110), urea (9.0), calcium carbonate (5.5), common salt (2.5), and a mineral‐vitamin supplement (2.5). The feed was offered in two equal meals at 07:00 and 17:00 h daily, with water available *ad libitum*. After a 14‐day adaptation period, rumen fluid was collected via a vacuum pump from multiple sites within the rumen prior to the morning feeding.

The collected material was immediately placed into insulated containers pre‐heated to 39°C to preserve microbial activity and transported to the laboratory under anaerobic conditions. In the lab, contents from both animals were pooled, thoroughly homogenized, and filtered through three layers of linen cloth under a continuous stream of CO_2_, as described by Rodrigues et al. (2020). The time from collection to the initiation of IVGP assay did not exceed 30 min to preserve microbial viability.

For IVGP parameters, 81 bottles were used across three separate runs. This included six treatments and four replicates per run, plus three blanks (rumen fluid only) per run, resulting in a total of 12 replicates per treatment (6 treatments × 4 replicates × 3 runs + 9 blanks = 81 bottles). For fermentation characteristics, 117 bottles were used across three runs. This included six treatments with six replicates per run, plus three blanks per run, resulting in a total of 18 replicates per treatment (6 treatments × 6 replicates × 3 runs + 9 blanks = 117).

Kinetics of GP were monitored over a 120 h incubation period, using a standardized protocol adapted from Marten and Barnes (1980). For the assay, 250 mg (DM basis) of the treated DPL substrate (ground to a 1 mm) was weighed into a 125 mL serum bottle. Each bottle received 5 mL of filtered rumen inoculum and 20 mL of anaerobic buffer solution (Marten and Barnes 1980). The bottles' headspaces were flushed with CO_2_ before sealing with butyl rubber stoppers and aluminum caps. After gentle mixing, the bottles were incubated at 39°C in a thermostatically controlled water bath. Cumulative GP was recorded at 3, 6, 9, 12, 16, 24, 48, 72, 96, and 120 h using a digital pressure system (Tracker 200; Baley and Mackey Ltd., Birmingham, UK), following the method of Theodorou et al. (1994). Gas pressure (psi) was used to calculate the corresponding volume (mL) based on the linear expression (Theodorou et al. 1994):

V=XP+I

where V is the volume (mL); P is the pressure (psi); X is the slope of the line of best fit; and I is the *y*‐intercept (a bias correction factor).

After 24 h of incubation, fermentation was terminated by placing the bottles in an ice bath. Once cooled to approximately 25°C (room temperature), final gas pressure was recorded. The pH of the fermentation contents was immediately measured using a calibrated pH meter (GLP22; Crison Instruments SA, Barcelona, Spain).

Subsamples of the supernatant were collected and preserved for subsequent analysis. A 5 mL sample was mixed with an equal volume of 0.1 N HCl and stored at −20°C for ammonia‐N determination. Another 5 mL sample was stabilized with an equal volume of 25% (w/v) metaphosphoric acid and frozen at −20°C for volatile fatty acids (VFAs) profiling. The remaining solid residues were prepared; one portion was allocated for rumen enzymatic activity analysis, and the other was dried in a forced‐air oven at 60°C for 48 h to determine in vitro DM digestibility (IVDMD).

### 2.8 Calculations

The IVGP were evaluated by fitting the data to the exponential model of Ørskov and McDonald (1979):

$$Y=a+b\left(1-e^{-\mathrm{ct}}\right)$$

where *Y* is the cumulative gas volume (mL) at time t (h); a is the gas volume from the soluble fraction (mL); *b* is the potential gas production from the insoluble but fermentable fraction (mL); and c is the fractional rate of gas production (h^−1^).

IVDMD was determined after 24 h of incubation. The contents of each bottle were filtered through pre‐weighed Whatman No. 1 filter paper and the residue dried in a forced‐air oven at 60°C for 48 h. IVDMD was calculated as:

$$\text{IVDMD}\;\left(g/\mathrm{kg}\;\mathrm{DM}\right)=\left[\left(\text{Initial}\;\mathrm{DM}\;\text{weight}—\text{Residual}\;\mathrm{DM}\;\text{weight}\right)/\text{Initial}\;\mathrm{DM}\;\text{weight}\right]\times 1000.$$

where initial DM weight is the weight of the substrate incubated, and residual DM weight is the dry weight of the residues post‐fermentation.

Metabolizable energy (ME) and in vitro organic matter (OM) digestibility (IVOMD) were estimated using the regression equations of Menke et al. (1979):

$$\mathrm{ME}=2.20+0.136\;\mathrm{GP}+0.0057\;\mathrm{CP}\;\left(g/\mathrm{kg}\;\mathrm{DM}\right).$$

$$\text{IVOMD}=148.8+8.89\;\mathrm{GP}+0.45\;\mathrm{CP}\;\left(g/\mathrm{kg}\;\mathrm{DM}\right)+0.65\;\mathrm{ash}\;\left(g/\mathrm{kg}\;\mathrm{DM}\right).$$

where GP is the net 24‐h gas production (mL/200 mg DM); CP and ash are the crude protein and ash contents (g/kg DM) of the original substrate, respectively.

Short‐chain fatty acids (SCFA) yield was calculated according to Getachew et al. (2002):

$$\text{SCFA}\;\left(\text{mmol}/g\;\mathrm{DM}\right)=0.0222\;\mathrm{GP}-0.00425.$$

where GP is the net 24‐h gas volume (mL/g DM).

Microbial protein synthesis (MPS) was estimated using the stoichiometric method of Blümmel et al. (1997):

$$\mathrm{MPS}\;\left(\mathrm{mg}/g\;\mathrm{DM}\right)=\mathrm{mg}\;\mathrm{ADS}-\left(\mathrm{mL}\;\mathrm{gas}\times 2.2\;\mathrm{mg}/\mathrm{mL}\right)$$

where ADS is the amount of apparently digested substrate (mg), gas is the net 24‐h gas production and 2.2 mg/mL is the stoichiometric factor for the mass of elements (carbon, hydrogen, and oxygen) used in SCFA production per mL of gas.

### 2.9 Ruminal Enzymatic Activity

Enzyme activities associated with the particulate matter fraction were assessed post‐fermentation. Solid pellets from centrifuged bottle contents were collected and processed according to a method adapted from Nogueira Filho et al. (2000). The pellets were re‐suspended in an equal volume of 0.1 M phosphate buffer (pH 6.8). To enhance cell disruption, lysozyme (4 g/L) and carbon tetrachloride (1 mL per 6 mL suspension) were added. The mixture was sonicated on ice (6 cycles of 30 s at 27,000 × *g* for 30 min at 4°C) and the resulting CS was carefully collected as the enzyme extract.

The activities of carboxymethyl cellulase (CMCase), microcrystalline cellulase (MCCase), and filter paper‐degrading (FPD) enzymes were determined as per Agarwal (2000). The CMCase assay mixture contained 1 mL of phosphate buffer (0.1 M, pH 6.8), 0.5 mL of the CS, and 0.5 mL of 1% carboxymethyl cellulose. The MCCase assay contained 1 mL of buffer, 1 mL of CS, and 1 mL of 1% microcrystalline cellulose. The FPD assay contained 1 mL of buffer, 1 mL of CS, and a 0.5 g strip of Whatman No. 1 filter paper. All assays were conducted at 39°C for 60 min. The reactions were terminated by adding 3 mL of 1% dinitrosalicylic acid reagent. The reducing sugars released were quantified colorimetrically as glucose equivalents using the method of Miller (1959). One unit (U) of enzyme activity was defined as the amount of enzyme required to release 1 μmol of glucose per hour per mL under the specified assay conditions.

### 2.10 Two‐Stage Nutrient Digestibility

A total of 135 bottles were used for the in vitro two‐stage digestion assay of the treated DPL substrates. This included six treatments, each with seven replicates, along with three blank controls (containing only rumen fluid) per run, across three independent experimental runs. The digestion procedure followed the two‐stage in vitro method originally outlined by Tilley and Terry (1963).

### 2.11 Laboratory Analysis

The contents of DM (method 930.15) ash (method 924.05) and nitrogen (N, method 954.01) content of the substrates were determined according to the standard methods of AOAC International (2012). The loss of DM was calculated by subtracting the initial weight of the substrate from the weight of the remaining residue after 21 days of incubation. Fiber components were analyzed using sequential detergent methods. Ash‐free neutral detergent fiber (NDFom) was determined according to van Soest et al. (1991) without sodium sulfite or α‐amylase. Ash‐free acid detergent fiber (ADFom) was analyzed following AOAC method 973.18 (2012). Acid detergent lignin (ADL), expressed as lignin(sa), was subsequently determined by treating the ADFom residue with 72% sulfuric acid, as described by Robertson and Van Soest (1981). Ammonia‐N concentration in the fermentation was measured colorimetrically according to Broderick and Kang (1980). The profile of VFAs was determined using gas–liquid chromatography, with ethyl‐butyric acid serving as the internal standard, following the procedure of Stewart and Duncan (1985). Thawed rumen fluid samples were transferred to 15 mL glass test tubes and centrifuged at 10,000 *g* for 10 min at 4°C (Mikro 220 R, Hettich, Germany). One mL aliquot of the upper phase was injected into a Varian 3400 gas chromatograph (Varian Inc., Walnut Creek, CA) equipped with an injector at 170°C, a flame‐ionization detector at 175°C, and a packed column (2 m × 2 mm i.d. glass containing 1–1965 10% SP‐1200/1% H_3_PO_4_ on 80/100 Chro‐mosorb W). The temperature of the gas chromatograph oven was isotherm and maintained at 140°C. Gas flow rates were nitrogen, 40 mL/min and compressed air, 300 mL/min.

### 2.12 Statistical Analysis

The experiment was arranged in a completely randomized design. Data for IVGP, fermentation parameters, nutrient disappearance, hydrolytic enzyme activity, and VFAs profiles were analyzed using the MIXED procedure of SAS (version 9.4) with the following model:

$$Y_{\mathrm{ijk}}=\mu +T_{i}+R_{j}+e_{\mathrm{ijk}}$$

where *Y*_*ijk*_ is the observed dependent variable, μ is the overall mean, T_i_ is the fixed effect of the *i* ^*th*^ treatment, *R*_*j*_ is the random effect of the *j*th experimental run, and *e*_*ijk*_ is the residual error.

Data of LiP activity and the chemical composition of the processed DPL were analyzed using the GLM procedure with the model:

$$Y_{\mathrm{ij}}=\mu +T_{i}+e_{\mathrm{ij}}$$

where *Y*_*ij*_ is the observed variable, μ is the overall mean, *T*_*i*_ is the fixed effect of the treatment, and *e*_*ij*_ is the residual error.

For both analyses, treatment means were compared using Duncan's multiple range test, and results were considered statistically significant at *p* < 0.05.

## 3 Results

### 3.1 LiP Activity

The LiP activity of the leopard moth gut bacterial mixture (comprising *Staphylococcus* sp., *Brevibacterium* sp., and *Enterobacter* sp.) cultured on DPL under different treatment conditions is presented in Table 1. No LiP activity was detected in either the control group (untreated DPL) or T1 (DPL supplemented with liquid medium only), both recording an activity of 0.00 U/mL/min. T2, which included the bacterial inoculum alone, exhibited a significantly higher activity of 0.202 U/mL/min (*p* < 0.01). The addition of glucose or urea enhanced LiP activity; T3 (with 0.5% glucose) and T4 (with 0.5% urea) showed increased activities of 0.269 and 0.266 U/mL/min, respectively, both significantly higher than T2 but not significantly different from each other. The highest LiP activity was observed in T5, which combined both 0.5% glucose and 0.5% urea along with the bacterial inoculum, reaching 0.328 U/mL/min. This value was significantly greater than all other experimental treatments (*p* < 0.05), indicating a synergistic effect of the combined carbon and nitrogen sources on enzyme production.

### 3.2 Changing Chemical Composition of Treated DPL

The chemical composition of DPL was significantly influenced by leopard moth gut bacterial mixture and the addition of growth stimulators (Table 2). DM weight loss increased significantly (*p* < 0.01) in all inoculated treatments compared to the control and T1. Compared to other treatments, the highest DM loss was observed in T5 (61.1 g/kg DM), indicating enhanced degradation when both glucose and urea were supplemented. CP content also increased significantly (*p* < 0.01) across the treated groups, rising from 52.2 g/kg in the control to 70.3 g/kg in T5, likely reflecting microbial protein enrichment. NDFom content decreased significantly (*p* = 0.04), with the greatest reduction again in T5 (571 g/kg DM) compared to control, T1, and T2, suggesting improved fiber degradation with combined nutrient supplementation. Although ADFom content showed a non‐significant decreasing trend (*p* = 0.09), its lowest value was recorded in T5 (409 g/kg DM). The ADL content decreased significantly (*p* < 0.01) in all inoculated treatments compared to the control and T1, with T5 achieving the lowest ADL (83.8 g/kg DM). In contrast, OM content remained statistically unchanged across treatments (*p* = 0.26), indicating that the overall proportion of organic constituents was largely preserved despite compositional shifts.

**TABLE 2:** Changing chemical composition (g/kg DM) of date palm leaf (DPL) treated leopard moth gut bacterial mixture (i.e., *Staphylococcus* sp., *Brevibacterium* sp., and *Enterobacter* sp.) with glucose and urea as growth stimulators.

Item	Experimental treatments						SEM	*p*
	Control	T1	T2	T3	T4	T5		
Weight loss (DM)	0.00^d^	14.7^c^	40.3^b^	46.5^b^	45.5^b^	61.1^a^	2.57	< 0.01
Organic matter	896	891	884	874	881	877	8.16	0.26
Crude protein	52.2^dc^	51.5^d^	60.2^bc^	65.2^ab^	68.2^ab^	70.3^a^	2.65	< 0.01
NDFom	596^a^	593^a^	589^a^	586^ab^	584^ab^	571^b^	4.75	0.04
ADFom	420	418	415	410	411	409	4.26	0.09
ADL	97.8^a^	94.3^a^	87.1^b^	84.7^b^	85.4^b^	83.8^b^	1.69	< 0.01

*Note:* In each row, values followed by different letters are significantly different (*p* < 0.05). The experimental treatments included control (untreated DPL), T1 (DPL + M9 liquid medium), T2 (DPL + bacterial inoculum comprising a mixture of *Staphylococcus* sp., *Brevibacterium* sp., and *Enterobacter* sp.), T3 (T2 + 0.5% glucose), T4 (T2 + 0.5% urea), and T5 (T2 + 0.5% glucose +0.5% urea).

Abbreviations: ADFom, ash‐free acid detergent fiber; ADL, acid detergent lignin; NDFom, ash‐free neutral detergent fiber; SEM, standard error of the mean.

### 3.3 IVGP, Fermentation Profile and Nutrient Digestibility

In vitro GP after 24 h of incubation (GP_24_) showed no statistically significant differences among treatments (*p* = 0.09; Table 3), although the highest value was observed in T5 (27.7 mL), and the lowest in the control group (21.5 mL). In contrast, total IVGP after 120 h was significantly affected by treatment (*p* < 0.01). The control and T1 had the lowest gas volumes (48.2 and 49.1 mL, respectively), whereas all other inoculated groups (T2–T5) exhibited significantly higher values, with T5 reaching the maximum at 61.2 mL. Similarly, GP from the fermentable fraction (b) increased significantly with bacterial inoculation (*p* < 0.01), ranging from 49.7 mL in the control to 63.5 mL in T5. However, the rate constant (c) of GP remained statistically unchanged across treatments (*p* = 0.23). The control and T1 had the lowest IVDMD values (385 and 388 g/kg DM, respectively), while T5 showed the highest disappearance at 453 g/kg (a 17.66% increase over the control), indicating enhanced microbial degradation. A similar trend was observed in IVOMD, which increased from 392 g/kg in the control to 452 g/kg in T5 (a 15.31% increase over the control). Estimated ME also improved significantly with treatment (*p* = 0.04), increasing from 4.83 MJ/kg DM in the control to 5.61 MJ/kg in T5. Concentration of SCFA tended to increase with treatment, although the difference was not statistically significant (*p* = 0.09). MPS was significantly elevated (*p* < 0.05) by treatment, increasing from 347 mg/g DM in the control to 405 mg/g in T5. While pH values decreased slightly across treatments, ranging from 6.37 in the control to 6.16 in T5, the changes were not statistically significant (*p* = 0.13). However, ammonia‐N concentration increased significantly with treatment (*p* = 0.03), rising from 8.85 mg/dL in the control to 10.2 mg/dL in T5.

**TABLE 3:** In vitro gas production (GP), fermentation parameters and two‐stage nutrient digestibility (g/kg DM) of date palm leaf (DPL) treated leopard moth gut bacterial mixture (i.e., *Staphylococcus* sp., *Brevibacterium* sp., and *Enterobacter* sp.) with glucose and urea as growth stimulators.

Item	Experimental treatments						SEM	*p*
	Control	T1	T2	T3	T4	T5		
GP_24_	21.5	22.1	25.8	26.5	25.1	27.7	1.88	0.09
Total GP	48.2^b^	49.1^b^	57.9^a^	59.2^a^	58.2^a^	61.2^a^	1.67	< 0.01
b	49.7^b^	51.1^b^	60.1^a^	61.3^a^	60.4^a^	63.5^a^	1.58	< 0.01
c	0.043	0.042	0.042	0.043	0.044	0.044	0.001	0.23
IVDMD	385^c^	388^c^	431^b^	444^ab^	440^ab^	453^a^	4.56	< 0.01
IVOMD	392^c^	399^c^	434^ab^	448^ab^	436^ab^	452^a^	7.81	< 0.01
ME	4.83^c^	4.90^bc^	5.35^abc^	5.45^ab^	5.32^abc^	5.61^a^	0.169	0.04
SCFA	1.88	1.94	2.27	2.33	2.21	2.43	0.140	0.09
MPS	347^c^	349^c^	385^b^	398^ab^	396^ab^	405^a^	5.66	< 0.01
pH	6.37	6.34	6.28	6.25	6.23	6.16	0.052	0.13
Ammonia‐N (mg/dL)	8.85^c^	8.95^bc^	9.57^abc^	9.90^ab^	10.1^a^	10.2^a^	0.309	0.03
Two‐stage digestibility								
Dry matter	391^c^	394^c^	441^b^	451^ab^	443^b^	461^a^	3.94	< 0.01
Organic matter	395^b^	411^b^	442^a^	455^a^	445^a^	459^a^	8.51	< 0.01
Crude protein	393^c^	406^bc^	437^ab^	436^ab^	449^a^	455^a^	9.98	< 0.01
NDFom	338^b^	343^b^	381^a^	383^a^	385^a^	393^a^	5.79	< 0.01
ADFom	330	337	344	346	348	351	6.59	0.09

*Note:* In each row, values followed by different letters are significantly different (*p* < 0.05). The experimental treatments included control (untreated DPL), T1 (DPL + M9 liquid medium), T2 (DPL + bacterial inoculum comprising a mixture of *Staphylococcus* sp., *Brevibacterium* sp., and *Enterobacter* sp.), T3 (T2 + 0.5% glucose), T4 (T2 + 0.5% urea), and T5 (T2 + 0.5% glucose +0.5% urea).

Abbreviations: ADFom, ash‐free acid detergent fiber; b, GP from the fermentable fraction (mL); c, fractional rate of GP (h^−1^); GP_24_, net gas production after 24 h of incubation (mL); IVDMD, in vitro dry matter (DM) disappearance (g/kg DM); IVOMD, in vitro organic matter disappearance (g/kg DM); ME, estimated metabolizable energy (MJ/kg DM); MPS, microbial protein synthesis (mg/g DM); NDFom, ash‐free neutral detergent fiber; SCFA, short chain fatty acid (mmol/g DM); SEM, standard error of the mean; Total GP, total gas production after 120 h of incubation (mL).

Two‐stage DM digestibility increased from 391 g/kg DM in the control to 461 g/kg in T5 (*p* < 0.01; Table 3), while OM digestibility rose from 395 g/kg DM to 459 g/kg over the same range (*p* < 0.01). CP digestibility also improved significantly (*p* < 0.01), with T5 exhibiting the highest value (455 g/kg DM), compared to 393 g/kg in the control. Similarly, NDFom digestibility increased from 338 g/kg DM in the control to 393 g/kg in T5 (*p* < 0.01). While ADFom digestibility showed an increasing trend (from 330 g/kg DM in the control to 351 g/kg in T5), the differences were not statistically significant (*p* = 0.09).

### 3.4 Ruminal Enzymatic Activity and VFAs

Treatment of DPL with a leopard moth gut bacterial mixture, alone or in combination with glucose and/or urea, significantly enhanced rumen hydrolytic enzyme activities (*p* < 0.01; Table 4). The activities of CMCase, MCCase, and FPD enzymes were all significantly higher in the inoculated treatments (T2–T5) compared to the control and T1. Activity of CMCase increased from 21.5 U/mL/min in the control to a maximum of 29.1 U/mL/min in T5. Similarly, MCCase activity rose from 14.2 U/mL/min in the control to 19.2 U/mL/min in T5, with all inoculated groups (T2–T5) showing significantly higher activity than the control. A comparable trend was observed in FPD activity, which increased from 17.3 U/mL/min in the control to 25.1 U/mL/min in T5. The inclusion of both glucose and urea (T5) consistently yielded the highest enzyme activities. In contrast, T1 (DPL with liquid medium only) did not differ significantly from the untreated control, confirming that the enhancements in enzymatic activity were attributable to the bacterial consortium and not the medium alone.

**TABLE 4:** Rumen hydrolytic enzyme activities (U/mL/min) of date palm leaf (DL) treated leopard moth gut bacterial mixture (i.e., *Staphylococcus* sp., *Brevibacterium* sp., and *Enterobacter* sp.) with glucose and urea as growth stimulators.

Item	Experimental treatments						SEM	*p*
	Control	T1	T2	T3	T4	T5		
Carboxymethyl cellulase	21.5^b^	22.3^b^	26.5^a^	27.3^a^	28.6^a^	29.1^a^	0.892	< 0.01
Microcrystalline cellulase	14.2^b^	14.5^b^	17.8^a^	18.3^a^	17.8^a^	19.2^a^	0.616	< 0.01
Filter paper degrading activity	17.3^b^	17.1^b^	22.8^a^	23.4^a^	23.6^a^	25.1^a^	1.49	< 0.01

*Note:* In each row, values followed by different letters are significantly different (*p* < 0.05). The experimental treatments included control (untreated DPL), T1 (DPL + M9 liquid medium), T2 (DPL + bacterial inoculum comprising a mixture of *Staphylococcus* sp., *Brevibacterium* sp., and *Enterobacter* sp.), T3 (T2 + 0.5% glucose), T4 (T2 + 0.5% urea), and T5 (T2 + 0.5% glucose +0.5% urea).

Abbreviation: SEM, standard error of the mean.

As shown in Table 5, total VFA concentrations increased significantly (*p* < 0.01) in all inoculated groups (T2–T5) compared to the control and T1. The highest VFA level was recorded in T5 (75.3 mmol/L), followed closely by T3 and T4 (73.4 and 73.3 mmol/L, respectively). A similar trend was observed for acetate concentrations, which rose significantly from 38.6 mmol/L in the control to 46.6 mmol/L in T5 (*p* < 0.01). All inoculated DPL exhibited higher acetate production than the control. Although the molar proportion of propionate, butyrate, valerate, isovalerate, and acetate to propionate ratio (A:P) was similar among the experimental groups (*p* > 0.05).

**TABLE 5:** Rumen volatile fatty acids (VFAs) concentration (mmol/L) of date palm leaf (DPL) treated leopard moth gut bacterial mixture (i.e., *Staphylococcus* sp., *Brevibacterium* sp., and *Enterobacter* sp.) with glucose and urea as growth stimulators.

Item	Experimental treatments						SEM	*p*
	Control	T1	T2	T3	T4	T5		
Total VFA	63.8^b^	64.5^b^	71.9^a^	73.4^a^	73.3^a^	75.3^a^	2.13	< 0.01
Acetate (A)	38.6^b^	39.1^b^	44.5^a^	45.1^a^	45.2^a^	46.6^a^	1.33	< 0.01
Propionate (P)	13.2	13.3	14.8	15.5	15.2	15.3	0.762	0.18
Butyrate	7.78	7.96	8.55	8.67	8.68	8.96	0.574	0.66
Valerate	1.51	1.56	1.52	1.50	1.58	1.61	0.055	0.64
Isovalerate	1.32	1.29	1.32	1.30	1.34	1.32	0.030	0.88
A:P	2.93	2.92	3.01	2.92	2.98	3.05	0.133	0.78

*Note:* In each row, values followed by different letters are significantly different (*p* < 0.05). The experimental treatments included control (untreated DPL), T1 (DPL + M9 liquid medium), T2 (DPL + bacterial inoculum comprising a mixture of *Staphylococcus* sp., *Brevibacterium* sp., and *Enterobacter* sp.), T3 (T2 + 0.5% glucose), T4 (T2 + 0.5% urea), and T5 (T2 + 0.5% glucose +0.5% urea).

Abbreviation: SEM, standard error of the mean.

## 4 Discussion

### 4.1 Activity of LiP

This study introduces a novel application of bacterial consortium *Staphylococcus* sp., *Brevibacterium* sp., and *Enterobacter* sp., isolated from the gut of the leopard moth for the nutritional enhancement of DPL. The results revealed that supplementing this consortium with readily metabolizable carbon (glucose) and nitrogen (urea) significantly boosted its ligninolytic potential. No LiP activity was detected in the uninoculated control or the medium only treatment (T1), confirming that enzymatic lignocellulose breakdown is strictly dependent on microbial inoculation. Treatments with glucose (T3) or urea (T4) individually induced a notable rise in enzyme activity. This aligns with established findings that simple carbohydrates and nitrogenous compounds can co‐induce ligninolytic enzyme expression by stimulating microbial proliferation and metabolic function (Li et al. 1994; Muhammed and Nambisan 2012). The highest LiP activity was observed with the dual addition of both substrates (T5), indicating that a synergistic interaction between carbon and nitrogen sources drives enzyme production. Such synergies have been previously reported in lignocellulose‐degrading white‐rot fungi and *actinobacteria* (Chen et al. 2025; Sharma and Arora 2015).

The efficacy of gut‐derived bacteria is well documented by literature. For instance, *Enterobacter hormaechei* PY12 and *Bacillus licheniformis* MX5, isolated from *Reticulitermes chinensis*, exhibited significant lignin‐degrading activity (Zhou et al. 2018). Similarly, Borji (2003) isolated *Enterobacter cloacae* , *Ochrobacterium anthropi*, and *Bacillus sphaericus* from *Anacanthotermes vagans*. More recently, *Bacillus* sp. BMP01 and *Ochrobactrumoryzae* BMP03 from the gut of *Cryptotermes* brevis were identified as effective agents for lignin and lignocellulose bioconversion (Tsegaye et al. 2019).

### 4.2 Chemical Composition

While termite gut bacteria have been explored for lignocellulose bioconversion (Azizi‐Shotorkhoft et al. 2016; Borji 2003), this is the first study to investigate microbial isolates from the leopard moth for improving the nutritive value of DPL. This bacterial consortium demonstrated substantial degradative activity, evidenced by decreased NDFom and ADL, alongside increased CP content and DM losses. These effects were most pronounced in T5, indicating intensified microbial action when glucose and urea were provided together, consistent with the mechanism reported for microbial consortia and white‐rot fungi (Brodeur et al. 2011; Crawford 1978).

The observed reduction in DM across bacterially treated samples indicated active microbial consumption of structural polysaccharides and lignin. The rise in CP content likely reflects microbial protein incorporation into the biomass, supporting earlier conclusions that biological treatments can enhance the nitrogen content of fibrous feeds (Asmare 2020; Shrivastava et al. 2012). Treatments T4 and T5 exhibited the highest CP concentrations, suggesting urea supplementation effectively stimulated bacterial growth and MPS. Although ADFom remained largely unchanged, the decrease in both NDFom and ADL in T5 implies partial disruption of lignin–cellulose linkages, a key prerequisite for improving fiber digestibility (van Soest 1994). The marked drop in ADL under combined glucose and urea supplementation supports the synergistic role in promoting ligninolytic activity, corroborating the enzyme assay data (Table 1). Comparable observations were made by Azizi‐Shotorkhoft et al. (2016), who found *Bacillus licheniformis* from *Microcerotermes diversus* most significantly altered the chemical composition of WS and DPL. Similarly, Borji (2003) reported that WS treated with a consortium of termite gut‐derived bacteria underwent more extensive chemical changes than the control.

### 4.3 In Vitro GP, Fermentation and Two Stage Nutrients Digestibility

The bacterial consortium significantly enhances IVGP and fermentation performance of DPL, especially when supplemented with carbon and nitrogen. Treatment T5 showed substantial increases in GP at 24 h (GP_24_), total GP (GP_120_), and fermentable gas fraction (b). This aligns with earlier findings that microbial pre‐treatment, particularly with ligninolytic species, enhances the degradation of fibrous residues (Sharma and Arora 2015). Improvement in IVDMD and IVOMD, especially in the T5 group, suggests a more effective breakdown of complex fiber components. These improvements are likely due to microbial‐driven partial breakdown of lignin, cellulose, and hemicellulose during incubation. This is supported by the decreases in ADL and NDFom (Table 2), reflecting a positive link between fiber degradation and digestibility (van Soest 1994). Increased levels of SCFAs yields and ME values in treated samples further indicate enhanced microbial fermentation efficiency, likely from the increased availability of fermentable carbohydrates (Menke and Steingass 1988).

The rise in MPS observed in T5 reflects greater microbial biomass generation, indicating improved nitrogen utilization and microbial proliferation (Blümmel et al. 1997). This may be associated with both the higher CP levels and reduced lignin content, which support more efficient microbial attachment and growth (Soliva et al. 2005). While rumen fluid pH remained stable, increased ammonia‐N concentrations in all treated groups, particularly in T5, point to better nitrogen availability from the supplemented CP content, which is crucial for microbial growth and effective rumen fermentation (Satter and Slyter 1974).

The two‐stage in vitro digestion trials confirmed that the bacterial inoculum from the leopard moth gut substantially improved the digestibility of DPL. Marked increases in DM and OM digestibility, especially in the T5 treatment, further support the role of microbial delignification in enhancing feed quality. Enhanced CP digestibility suggests successful protein enrichment, likely driven by microbial biosynthesis and improved solubilization in the presence of urea. Urea, acting as a nitrogen source, may have stimulated microbial activity and enzymatic protein degradation (van Soest 1994). The improved digestibility of NDFom indicates efficient disruption of plant cell walls consistent with the observed reductions in ADL. The superior performance of T5 supports the hypothesis that combined supplementation with glucose and urea promotes bacterial colonization and the secretion of fiber‐degrading enzymes (Chen et al. 2025; Okano et al. 2009). Supporting evidence includes the work of Kumawat et al. (2025), who reported enhanced IVDMD following inoculation with fibrolytic bacteria from *Coptotermes heimi*. Overall, the success of such microbial treatments depends on factors like fermentation duration, feedstock type, and the inclusion of growth enhancers like glucose or urea (Borji 2003).

### 4.4 Rumen Enzyme Activity and VFAs

While hydrolytic enzymes are crucial indicators of the microbial attachment and substrate utilization (Nogueira Filho et al. 2000), few studies have fully investigated the ruminal enzymatic response to biologically pretreated by‐products. In this study, the marked increase in CMCase, MCCase, and FPD enzyme activities, particularly in the T5, indicates that supplementing the inoculum with glucose and urea creates an optimal environment for microbial colonization and enzyme production. This elevated enzyme activity correlates with the observed lignin breakdown (Table 2), enhanced IVGP (Table 3), and improved digestibility (Table 3), demonstrating that microbial treatment not only modifies the feed's chemical structure but also boosts its fermentative properties. Specifically, the increased FPD activity reflects overall cellulolytic capacity, supporting the enhancements in NDFom digestibility. Moreover, the increased production of total VFA and acetate in the T5 confirms that the combined addition of an energy source (glucose) and a nitrogen source (urea) enhances microbial fermentation. This is consistent with previous research showing that fermentable carbohydrates and non‐protein nitrogen compounds stimulate microbial activity and fiber degradation, thereby promoting VFA production during in vitro fermentation (Getachew et al. 2004; van Soest 1994).

Interestingly, despite the rise in total VFA and acetate, the concentrations of propionate, butyrate, valerate, or isovalerate remained unchanged. This suggests that while fermentation intensity increased, the overall fermentation profile was stable. The unchanged acetate‐to‐propionate ratio, coupled with higher total VFA production, indicates a shift favoring acetate generation without compromising propionate levels, an outcome beneficial for both fiber digestion and rumen function.

### 4.5 Integrated Mechanisms of Enhanced Fermentation

The significant improvements in the nutritive value and fermentative characteristics of DPL result from a cascade of interconnected biological and chemical mechanisms, initiated by the leopard moth gut bacterial consortium and amplified by microbial growth simulators. The foundational mechanism is the enzymatic deconstruction of the recalcitrant lignocellulosic matrix of DPL. The bacterial consortium secreted LiP, a potent oxidative enzyme that depolymerizes the lignin barrier encrusting cellulose and hemicellulose. This unlocking of digestible carbohydrates is directly evidenced by the significant reduction in ADL and NDFom, most profoundly in T5 (Table 2). Subsequently, the consortium's cellulolytic enzymes, specifically CMCase, MCCase, and FPD enzymes (Table 4), hydrolyzed the exposed cellulose and hemicellulose into fermentable sugars, a critical pre‐digestion step that bypasses the rate‐limiting lignin breakdown for rumen microorganisms.

The multi‐strain consortium operated on a principle of metabolic synergy. The three strains likely possess complementary enzymatic profiles, engaging a division of labor where some excelled at lignin degradation and others at cellulolysis. This collective action created a robust enzymatic system capable of tackling the complex heterogeneity of the DPL more effectively than any single strain. Cross‐feeding of metabolic byproducts likely created a self‐reinforcing cycle of degradation. The glucose and urea optimized the growth environment, supercharging the degradation process; glucose provided a readily available carbon source, priming bacterial metabolism for rapid proliferation and the synthesis of expensive extracellular enzymes. Urea supplied essential nitrogen for synthesizing amino acids, proteins, and the enzymes. The combination of glucose and urea in T5 provided both the energy and the fundamental building blocks for microbial growth, leading to a synergistic explosion in metabolic activity. This might explain the highest LiP activity, greatest DM loss, and most significant compositional improvements. The biological pre‐treatment created a superior substrate for the rumen microbiota. The delignified and partially hydrolyzed DPL presented a larger surface area and fewer physical barriers, allowing for faster and more extensive colonization and fermentation, as reflected in the higher IVGP and rate of gas production. The increase in CP content (Table 2) provided additional nitrogen for rumen microbes, leading to higher MPS and ammonia‐N concentrations. The pre‐hydrolysis of structural carbohydrate created a pool of readily fermentable sugars, leading to rapid gas production and higher SCFA concentrations.

In summary, the enhancement is a sequential cascade: the bacterial consortium, amplified by glucose and urea, produced a potent cocktail of ligninolytic and cellulolytic enzymes. This enzymatic action transformed the DPL into a more accessible, digestible, and nitrogen‐rich substrate. This provided an ideal environment for the rumen microbiome, leading to significant enhancements in gas production, nutrient digestibility, and microbial fermentation efficiency.

## 5 Conclusion

This study successfully demonstrates a novel biotechnological approach for enhancing the nutritional value of DPL. This finding confirms that a lignocellulose‐degrading bacterial consortium (comprising *Staphylococcus* sp., *Brevibacterium* sp., and *Enterobacter* sp.) derived from the gut of the leopard moth ( *Zeuzera pyrina* L.) can effectively degrade lignocellulose, with its activity significantly amplified by supplementation of glucose and urea. The T5, which combined the microbial inoculum with both carbon and nitrogen sources, yielded the most pronounced improvements, as evidenced in IVGP, nutrient digestibility, MPS, and rumen enzyme activity. This synergistic effect underscores the critical role of optimizing growth conditions for optimizing the efficiency of biological pre‐treatments. By leveraging insect gut microbiota and simple nutrient supplements, this research provides a sustainable and innovative strategy to convert low quality biomass into a valuable feed resource for ruminants. This approach holds significant potential to lower feed costs, improve resource efficiency, and promote more environmentally sound livestock production. To translate these promising in vitro results into practical benefits, future research should focus on in vivo feeding trials to validate the finding and explore the scalability of this process for real‐world application.

## Author Contributions

**Afrooz Sharifi:** conceptualization (equal), data curation (equal), project administration (equal), supervision (lead), writing – original draft (lead). **Ayoub Azizi:** conceptualization (equal), data curation (equal), writing – review and editing (equal). **Ali Kiani:** conceptualization (equal), data curation (equal), writing – review and editing (equal).

## Funding

The authors have nothing to report.

## Ethics Statement

All animal‐related procedures in this study were carried out in accordance with the ethical principles outlined by the Federation of Animal Science Societies (FASS 2010) for the responsible care and use of agricultural animals in research. The experimental protocol received prior approval from the Animal Ethics Committee of the Faculty of Agriculture at Lorestan University, ensuring adherence to established animal welfare standards.

## Conflicts of Interest

The authors declare no conflicts of interest.

## Data Availability

Data will be made available on request.
